# The protective effect of Luteolin on chicken spleen lymphocytes from ammonia poisoning through mitochondria and balancing energy metabolism disorders

**DOI:** 10.1016/j.psj.2023.103093

**Published:** 2023-09-09

**Authors:** Dechun Chen, Fanyu Shen, Jiahao Liu, Haojinming Tang, Kai Zhang, Xiaohua Teng, Falong Yang

**Affiliations:** ⁎ Southwest Minzu University Key Laboratory of Animal Medicine in Sichuan Province, Southwest Minzu University, Chengdu 610041, China; † College of Animal Science and Technology, Northeast Agricultural University, Harbin 150030, China

**Keywords:** ammonia, chicken, Luteolin, mitochondria, energy metabolism

## Abstract

Ammonia poses a significant challenge in the contemporary intensive breeding industry, resulting in substantial economic losses. Despite this, there is a dearth of research investigating efficacious strategies to prevent ammonia poisoning in poultry. Consequently, the objective of this study was to investigate the molecular mechanisms through which Luteolin (**Lut**) safeguards mitochondria and restores equilibrium to energy metabolism disorders, thereby shielding chicken spleen lymphocytes from the detrimental effects of ammonia poisoning. Chicken spleen lymphocytes were categorized into 3 distinct groups: the control group, the ammonia group (with the addition of 1 mmol/L of ammonium chloride), and the Lut group (with the treatment of 0.5 μg/mL of Lut for 12 h followed by the addition of 1 mmol/L of ammonium chloride). These groups were then cultured for a duration of 24 h. To investigate the potential protective effect of Lut on lymphocytes exposed to ammonia, various techniques were employed, including CCK-8 analysis, ultrastructural observation, reagent kit methodology, fluorescence microscopy, and quantitative real-time PCR (**qRT-PCR**). The findings indicate that Lut has the potential to mitigate the morphological damage of mitochondria caused by ammonia poisoning. Additionally, it can counteract the decline in mitochondrial membrane potential, ATP content, and ATPase activities (specifically Na^+^/K^+^-ATPase, Ca^2+^-ATPase, Mg^2+^-ATPase, and Ca/Mg^2+^-ATPase) following exposure to ammonia in lymphocytes. Lut also has the ability to regulate the expression of genes involved in mitochondrial fusion (Opa1, Mfn1, and Mfn2) and division (Drp1 and Mff) in spleen lymphocytes after ammonia exposure. This regulation leads to a balanced energy metabolism (HK1, HK2, LDHA, LDHB, PFK, PK, SDHB, and ACO2) and provides protection against ammonia poisoning.

## INTRODUCTION

Ammonia, a highly prevalent noxious gas in the poultry farming industry, poses a significant threat due to its ease of production. Exposure of chickens to ammonia leads to detrimental tissue damage, specifically affecting the thymus (Chen et al., 2020a), spleen (Chen et al., 2020b), and trachea (Wang et al., 2020). Consequently, these adverse effects result in substantial economic losses within the poultry breeding sector. The spleen, an immune organ in the human body, exhibits resistance against detrimental external stimuli. The exposure to ammonia poisoning induces alterations in the microstructure of chicken spleen tissue, as well as disruptions in ATPase activity, thereby resulting in disorders in energy metabolism. Consequently, this leads to inflammatory damage and procedural necrosis in the chicken spleen tissue (An et al., 2019). Consequently, the investigation of efficacious strategies to safeguard against or prevent the toxic harm inflicted by ammonia assumes paramount importance for the advancement of poultry farming and human well-being.

Luteolin (**Lut**) is a naturally occurring flavonoid compound that is present in a variety of plants. Its biological activities include anti-inflammatory, antioxidant, and cytotoxic effects, which are mediated through in vivo anti-inflammatory and antioxidant mechanisms. These mechanisms have been shown to effectively mitigate cellular damage (Ajibade et al., 2022; Caporali et al., 2022). Previous research has demonstrated that Lut exhibits the potential to mitigate the presence of inflammatory mediators through the elimination of free radicals, thereby offering therapeutic benefits in the treatment of carbon tetrachloride (Habibi et al., 2021), mercury (Tan et al., 2018), arsenic (Quiller et al., 2018), and lead (Baty et al., 2020) poisoning. Additionally, Lut has been shown to enhance energy supply and exert a protective role (Zhang et al., 2022). It is worth noting that mitochondrial dysfunction can disrupt the equilibrium of energy metabolism, leading to the manifestation of energy metabolism disorders (Gore et al., 2022). ATPase plays a crucial role in facilitating the hydrolysis of ATP, resulting in the liberation of a substantial quantity of energy. This process holds significant importance in cellular material transportation and energy metabolism.

This study aims to investigate the effects of Lut intervention on chicken spleen lymphocytes exposed to ammonia, specifically focusing on changes in mitochondria and energy metabolism. These changes will be assessed through the determination of ATP content and ATPase activities, mitochondrial membrane potential, expression of mitochondrial dynamics-related genes (Drp1, Opa1, Mfn1, Mfn2, and Mff), energy metabolism-related genes (HK1, HK2, LDHA, LDHB, PFK, PK, SDHB, and ACO2), miR-155-5p, and relative mRNA expression of the PI3K/AKT pathway. The objective of this study is to examine the effects of Lut on mitochondria and energy metabolism following exposure of spleen lymphocytes to ammonia. The findings of this research can contribute to the development of strategies and experimental evidence for mitigating ammonia poisoning in poultry during animal husbandry practices.

## MATERIALS AND METHODS

### Ethics Statement

The present study was conducted in strict adherence to the guidelines set forth by the Institutional Animal Care and Use Committee of Southwest Minzu University (SWUN-MR0056) and the Regulations for the Administration of Affairs Concerning Experimental Animals of the State Council of the People's Republic of China.

### Primary Splenic Lymphocyte Culture

Primary splenic lymphocytes were obtained from 45-day-old Ross chickens. After collecting cardiac blood and euthanizing the chickens, they were immersed in a 0.2% benzalkonium bromide solution for 10 min. The spleens were then gently pressed and pushed through a sterile mesh with a pore size of 70 μM. Lymphocytes were separated using lymphocyte separation medium from TianJin Haoyang Biological Manufacture Co. Ltd., China. The operational procedure followed the guidelines outlined in the reference by Chen et al. (2020b). The cells were cultured using RPMI 1640 medium supplemented with 10% fetal bovine serum (Bioind, Israel) and 1% antibiotic antifungal solution (Hyclone, New Zealand). The splenic lymphocytes were adjusted to a density of 1.0 × 10^7^ cells/mL in a 6-well plate and incubated at 37°C and 5% CO_2_.

### CCK-8 Detection of Cell Vitality

The protection concentration of Lut in chicken spleen lymphocytes exposed to ammonium chloride was determined using CCK-8, a medium commonly employed in vitro to simulate ammonia exposure (Chen et al., 2020b). A 96-well plate was coated with 20 μg/mL polylysine overnight, after which splenic lymphocytes were added and cultured for 3 h to facilitate cell adhesion to the plate. The lymphocytes were then cultured in Lut medium with final concentrations of 0.01 μg/mL, 0.5 μg/mL, 1.0 μg/mL, 5.0 μg/mL, and 25 μg/mL. After a period of 12 h, the culture medium was discarded. The ammonium chloride (**NH**_**4**_**Cl**) group and Lut group were then supplemented with 1 mmol/L of NH_4_Cl for a duration of 24 h. The concentration of NH_4_Cl was determined based on the findings of previous research conducted by Chen et al. (2020b). The control group, on the other hand, received only the culture medium. Subsequently, each pore was treated with 10 μL of CCK-8 and 100 μL of medium in a dark environment. Following an incubation period of 3 h, the absorbance of the cells at a wavelength of 490 nm was measured using an enzyme-labeling instrument (318MC, Shanghai Sanke Instrument Co., Ltd., Shanghai, China). The cell viability was quantified and the concentration of the Lut-treatment groups was determined.

### Ultrastructural Observation

Cells were inoculated onto a 96-well plate containing 2 mL of cell suspension at a concentration of 1.0 × 10^7^ cells/mL for ultrastructural observation. Following a stable cell culture period of 3 h, the Lut group was supplemented with Lut, while the control group and NH_4_Cl group received an equivalent volume of culture medium. The plate was then placed in an incubator and incubated for 12 h. Subsequently, each group was treated with a 1 mmol/L NH_4_Cl solution and incubated for an additional 24 h to facilitate subsequent experiments.

The treated cells were collected in a centrifuge tube and subjected to centrifugation at a speed of 1,500 r/min for a duration of 10 min. The resulting supernatant was discarded. To resuspend the cells, a 0.5% glutaraldehyde fixed solution was employed. Subsequently, the cells were transferred to a new EP tube and subjected to centrifugation. For fixation, a 3% glutaraldehyde fixing solution was utilized. The cells were then rinsed with phosphate buffer and fixed with osmic acid. The subsequent steps involved dehydration with acetone, impregnation with epoxy resin and hardener, embedding, slicing, ultrathin sections at a thickness of 40 to 50 nm, and staining with uranium acetate. Transmission electron microscopy (**TEM**) was employed to observe the ultrastructural changes.

### The Determination of ATP Content and ATPase Activities

#### The Detection of ATP Content

The cells were centrifuged and collected to facilitate the detection of ATP content. Subsequently, 500 μL of hot double distilled water was added to EPs, which were then placed in a hot water bath. The cells were homogenized and crushed using a glass homogenizer. The resulting cell suspension was heated at 100°C for 10 min in boiling water. Afterward, the cells were mixed and subjected to repeated blows for 1 min. Finally, the homogenate was used to detect ATP content following the instructions provided by the reagent kit (Nanjing Jiancheng Reagent Kit, Nanjing, China).

#### The Determination of ATPase Activities

Cultured cells were harvested and subjected to centrifugation. Following removal of the supernatant, 0.5 mL of extraction solution was added to the sediment and thoroughly mixed. Ultrasound fragmentation was employed to create a cell suspension. The activities of ATPase enzymes (specifically Na^+^/K^+^-ATPase, Ca^2+^-ATPase, Mg^2+^-ATPase, and Ca/Mg^2+^-ATPase) were assessed using the reagent kit instructions (Nanjing Jiancheng Reagent Kit, Nanjing, China). The protein content in the cells was determined using the BCA method.

### Mitochondrial Membrane Potential Assay

Following the cultivation process, the cells were gathered in centrifuge tubes for the purpose of centrifugation. The resulting supernatant was discarded, and the cells were subsequently resuspended in 0.5 mL of JC-1 working solution (Biyuntian Reagent Kit, China). The resuspended cells were then incubated in an incubator for a duration of 15 min, after which they underwent centrifugation. The resuspension cells were subjected to an additional centrifugation step with 2 mL of PBS buffer in order to eliminate the supernatant, which was repeated once. Finally, the cells were resuspended in 0.3 mL of PBS and examined using a fluorescence microscope (Nikon, Japan). The software program IMAGE J (V1.8, National Institutes of Health, Bethesda, MD) was utilized for the quantification of red/green fluorescence intensity and the subsequent calculation of the red/green fluorescence ratio. A total of 3 fields of view were randomly chosen for the determination of the respective percentages.

### Relative miRNA and mRNA Expression

Total RNA was extracted from lymphocytes using Trizol reagent (TaKaRa, Japan). Complementary DNA (**cDNA**) was synthesized following the instructions provided by the manufacturer (Roche, Shanghai, China). U6 and β-actin were utilized as internal references for mRNA. The primer sequences for U6, miR-155-5p, β-actin, mitochondrial dynamics genes (Drp1, Opa1, Mfn1, Mfn2, and Mff), energy metabolism (HK1, HK2, LDHA, LDHB, PFK, PK, SDHB, and ACO2), and the PI3K/AKT pathway were synthesized by Sangon Biotech Co., Ltd. (Shanghai, China) (Table 1). The qRT-PCR analysis was conducted using a LightCycler 480 II Detection System (Roche, Switzerland). The melting curve analysis revealed a single peak for each PCR product. The 2^−ΔΔCt^ method was employed to determine the relative levels of mRNAs in the duodenum, cecum, and colon tissues (Li et al., 2023).

**Table 1:** Primer sequences.

Gene	Primer sequence (5′–3′)	GenBank no.
β-Actin	F: CCGCTCTATGAAGGCTACGC R: CTCTCGGCTGTGGTGGTGAA	
Drp1	F: TCGTGCTCCTCCTGGTGTTCC R: TTCTGTGCGTTGCCACCGATG	XM_046923870
Opa-1	F: -CGGTTGCGAGAGCTTGACAGG R: TTCATTGTCTGTGCTGCTGGAAGG	XM_046923870.1
Mfn1	F: GAGGTGGAGGCTGTCGGATGG R: AGAGGTGAGCAGAGGTG GAAGTAC	XM_046923917.1
Mfn2	F: CCTCTCCTCCTGCCTG GAACTG R: CCTGGGTTTCAGAAGTGGC	XM_040689233.2
Mff	F: ACGGTGTGCCAGGTTAGTTATGC R: GTCCGAGCCTGAGCCG ATTAAC	CP100563.1
HK1	F: CATGTCGGTGCCGCAGAAGTC R: GAAGGTGCTCACAAGAC AGACTCG	XM_046919714.1
HK2	F: TGGAGGTGAAGCGGAGGATGAG R: GCACCAGCAGCACACGGAAG	NM_001396482.1
LDHA	F: TGCCTGTCTGGAGCGGAGTG R: GTCCACCACCTGCTTGT GAACC	NM_205284.2
LDHB	F: GCAGGTGTTCGTCAGCAAGAGG R: GGCAGGCCACTCAACTT CCATG	XM_040698131.2
PFK	F: ATCAGTGAGGAGGTGGCGAAGG R: CATGTCGGTGCCGCAGAAGTC	AB083368.1
ACO2	F: GCCAAGGACATAAACCAGGA R: TATGAGTCTGTGCCAATCAAC	NM_204188.3
SDHB	F: TGGACGGACTCTATGAGTGCATCC R: TTGAAGTTGTGCCAGGCGTTCC	XM_048967462.1
PI3K	F: GGAATGAATGGCTGTCGTATGAC R: CCAATGGACAGTGCTCCTCTTTA	XM_046923914.1
AKT	F: AAGGAAGGATGGCTCCACAAA R: CGTTCCTTGTAGCCAATGAATGT	NM_001396387.1
U6	CACGCAAATTCGTGAAGCGTTCCA	
miR-155-5p	TCGCAGCGCTATTGCACATTACTAAG	

### Statistical Analysis

The statistical significance of the detection kit results and mRNA expression data were assessed through the utilization of multiple factor analysis of variance (**ANOVA**) and Tukey's multiple comparison analysis. GraphPad Prism (version 8.0, Graph Pad Software Inc., San Diego, CA) was employed for the statistical analysis of all data. The results were presented as means ± SD (*n* = 5). A significance level of *P* < 0.05, denoted by *, was deemed statistically significant. A significance level of *P* < 0.01, denoted by **, was considered statistically highly significant. A significance level of *P* < 0.001, denoted by ***, was considered statistically extremely significant. On the contrary, a result that was not statistically significant, denoted as ns (*P* > 0.05).

## RESULTS

### Cell Viability Assay

**Figure 1:**
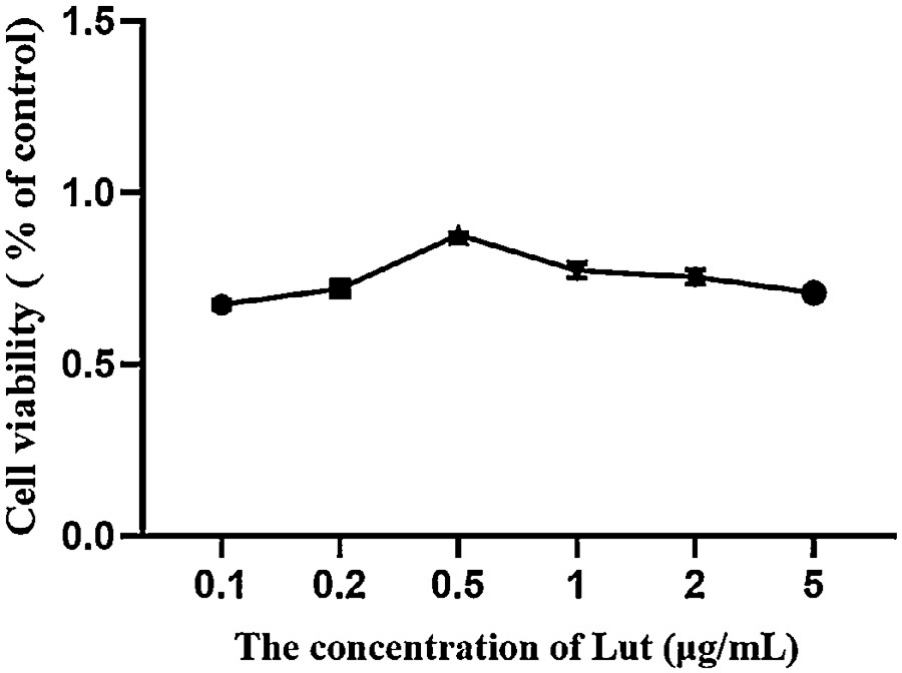
Cell viability assay of Lut on spleen lymphocytes in chickens exposed to ammonia.

The figure presented in Figure 1 illustrates the results of the cell viability test conducted on chicken spleen lymphocytes that were subjected to various concentrations of Lut, following damage caused by NH_4_Cl. The cell viability percentages for lymphocytes treated with Lut concentrations of 0.1, 0.2, 0.5, 1, and 5 μg/mL, in combination with NH_4_Cl for a duration of 24 h, were found to be 68.2, 72.1, 89.5, 77.3, 75.5, and 70.8%, respectively, across different experimental groups. Among these concentrations, the Lut group was selected based on a concentration of 0.5 μg/mL.

### Ultrastructural Observation

**Figure 2:**
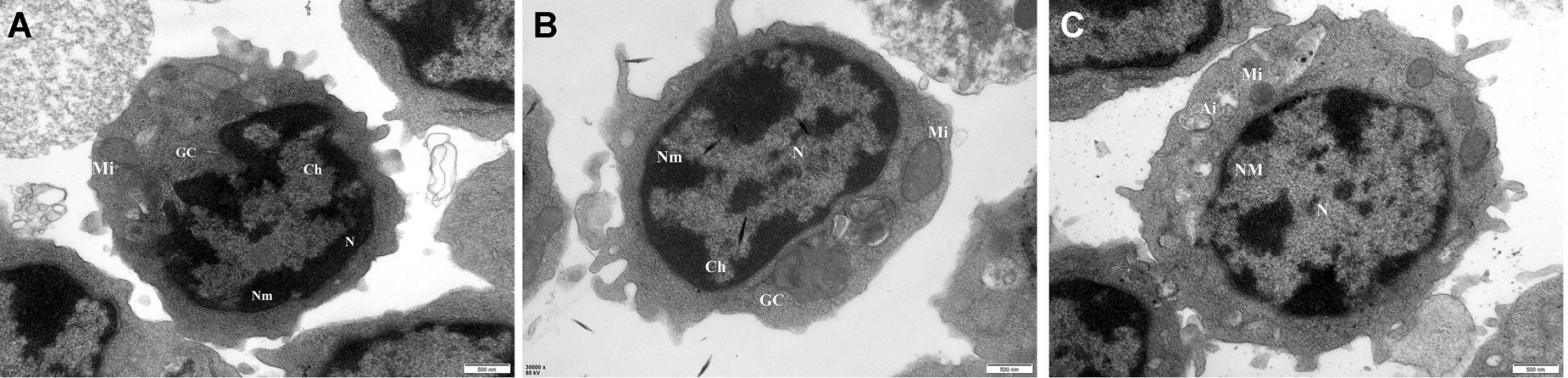
Effect of Lut on the ultrastructure changes in chicken spleen lymphocytes exposed to ammonia (×30,000). (A) The control group; (B) NH_4_Cl group; (C) Lut group. nuclei (N); nuclear membrane (NM); nucleolus (No); chromatin (Ch); mitochondria (Mi); Golgi apparatus (GC); autophagosomes (Ai); (N); autophagosomes (Au).

To examine the impact of Lut on lymphocyte damage induced by ammonia exposure, the ultrastructural alterations were investigated using TEM technology. The cellular ultrastructure of each group was observed, with the control group (Figure 2A) exhibiting a normal-shaped nucleus (**N**), intact bilayer nuclear membrane (**NM**) structure, oval nucleolus (**No**), evenly distributed chromatin (**Ch**), clearly visible mitochondria (**Mi**) cristae, and overall normal morphology. In the NH_4_Cl group (Figure 2B), aberrant nuclear morphology was observed, characterized by the collapse of the double nuclear membrane (**NM**), loss of nuclear integrity, severe collapse and unclear appearance of the nuclear membrane, disappearance of the nucleolus, and accumulation of chromatin beneath the nuclear membrane. Additionally, a reduction in the number of mitochondria was noted, accompanied by a significant disruption of mitochondrial cristae. The morphology and structure of the Golgi apparatus (**GC**) have undergone alterations, characterized by the presence of autophagosomes (**Au**) and autophagosomes (**Ai**) containing a substantial amount of content. In the LUT group (Figure 2C), certain nuclei (**N**) exhibited deformities, with partial disappearance of the double nuclear membrane (**NM**) structure and the emergence of wrinkles. Additionally, a small proportion of cell nucleoli vanished. The reduced number of mitochondria led to the rupture of mitochondrial spines and subsequent swelling. Infrequently, Au and Ai were observed.

### Lut Enhanced ATP Content and ATPase Activities After Ammonia Exposure

**Figure 3:**
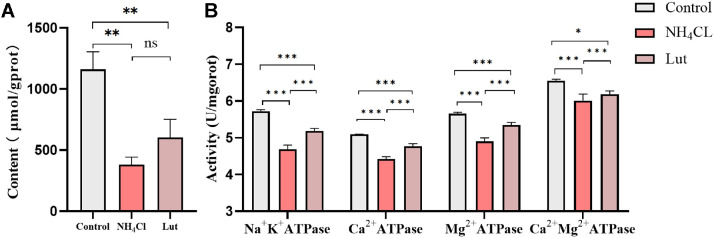
ATP content and ATPases activities (A) ATP content; (B) ATPases activities.

The reagent kit method was employed in this experiment to detect ATP content and ATPase activities. The study aimed to investigate the impact of Lut on ATP content in chicken spleen lymphocytes induced by ammonia exposure, as depicted in Figure 3.

#### Lut Increased ATP Content After Ammonia Exposure

The figure presented in Figure 3A demonstrates the detection of ATP content. Statistical analysis revealed a significant decrease in ATP content in both the NH_4_Cl and Lut groups compared to the control group (*P* < 0.01). Furthermore, when comparing the NH_4_Cl group to the Lut group, there was a nonsignificant increase in ATP content (*P* > 0.05).

#### Lut Increased ATPase Activities After Ammonia Exposure

The impact of Lut on the ATPase activities of chicken spleen lymphocytes, induced by exposure to ammonia, is illustrated in Figure 3B. In comparison to the control group, the Na^+^-K^+^-ATPase, Ca^2+^-ATPase, Mg^2+^-ATPase, and Ca^2+^-Mg^2+^-ATPase activities in the NH_4_Cl group exhibited a highly significant decrease (*P* < 0.001). Conversely, the 4 ATPase activities in the Lut group were significantly diminished when compared to the control group (*P* < 0.05). Furthermore, the 4 ATPases in the Lut group displayed a significant increase in comparison to the NH_4_Cl group (*P* < 0.001). The findings from the analysis of ATPase activities suggest that Lut has the potential to mitigate energy metabolism disruptions resulting from ammonia-induced damage.

### Protective Effect of Lut on Mitochondrial Damage in Lymphocytes Exposed to Ammonia

**Figure 4:**
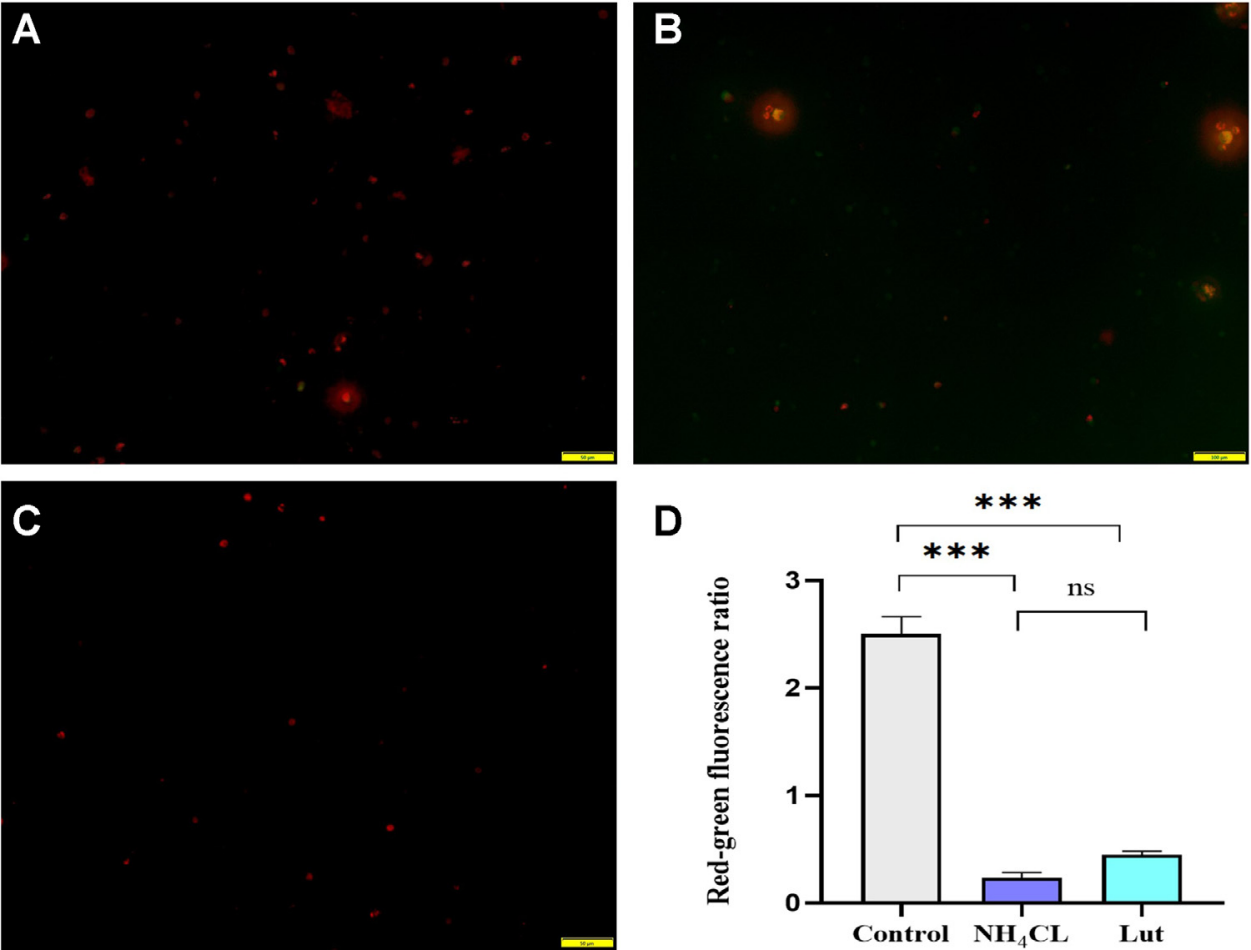
Effect of Lut on mitochondrial membrane potential of lymphocytes exposed to ammonia (×400). (A) The control group; (B) NH_4_Cl group; (C) Lut group; (D) Quantitative analysis of value of red/green fluorescence.

In this experiment, the JC-1 fluorescent probe was employed to assess the mitochondrial membrane potential and ascertain the protective impact of Lut on mitochondrial impairment in lymphocytes exposed to ammonia, as depicted in Figure 4. The NH_4_Cl group (Figure 4B) exhibited an elevated quantity of green monomers relative to the control group (Figure 4A), whereas the Lut group (Figure 4C) displayed a reduced quantity of green monomers compared to the NH_4_Cl group. The analysis of the red/green fluorescence ratio is presented in Figure 3D. The control group exhibited a mean value of 2.35, while the NH_4_Cl treatment group had a mean value of 0.235 and the Lut group had a mean value of 0.448. The observed disparity between the control group and both the NH_4_Cl and Lut groups was found to be highly significant (*P* < 0.001). These findings suggest that Lut possesses the ability to mitigate NH_4_Cl-induced mitochondrial damage in lymphocytes.

### Lut Alleviated Mitochondrial Dysfunction Disorders Caused by Ammonia Exposure

**Figure 5:**
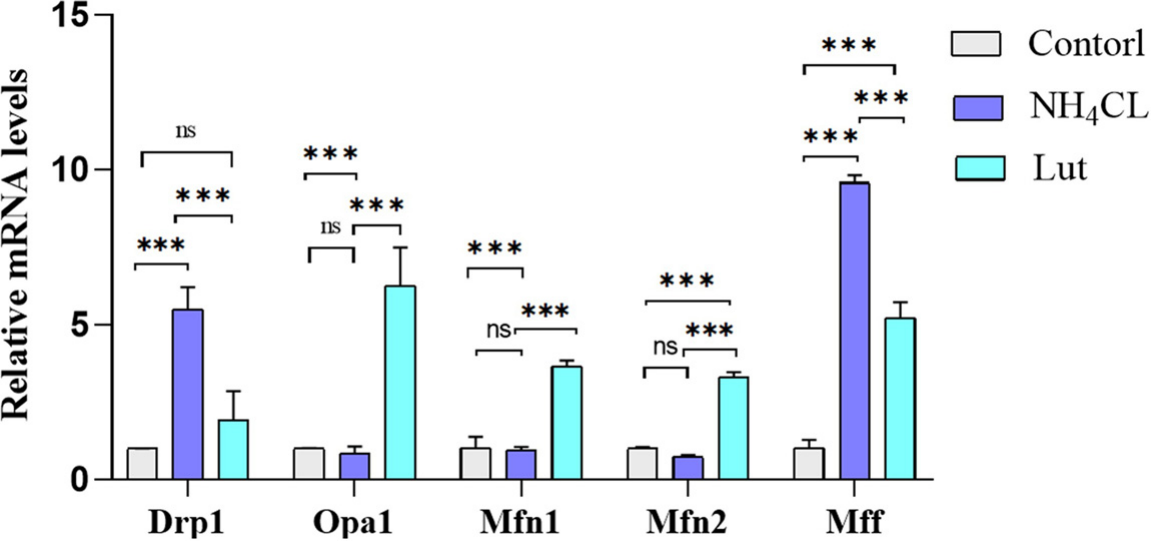
Results of qRT-PCR for mitochondrial dynamics-related genes (Drp1, Opa1, Mfn1, Mfn2, and Mff).

qRT-PCR was employed to assess the mRNA expression of genes associated with mitochondrial dynamics, as depicted in Figure 5. In comparison to the control group, the NH_4_Cl group exhibited a significant increase (*P* < 0.05) in the relative mRNA expression levels of Drp1, Opa1, Mfn1, and Mff, while the relative mRNA expression levels of Mfn2 increased but not significantly (*P* > 0.05). Conversely, the Lut group demonstrated a significant reduction (*P* < 0.01) in the mRNA relative expression of Drp1, Opa1, and Mff, whereas the mRNA relative expression of Mfn1 and Mfn2 decreased, albeit not significantly (*P* > 0.05), when compared to the NH_4_Cl group. The findings suggest that Lut has the potential to mitigate mitochondrial dysfunction induced by ammonia, thus ameliorating disturbances in energy metabolism.

### Lut Alleviated Energy Metabolism Disorders Caused by Ammonia Exposure

**Figure 6:**
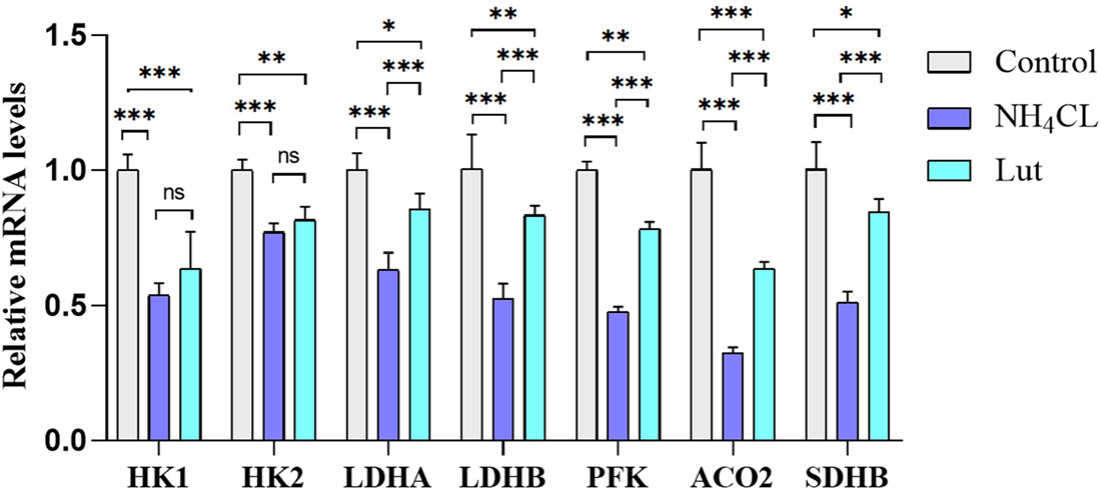
Results of qRT-PCR for energy metabolism (HK1, HK2, PFK, LDHA, LDHB, ACO2, and SDHB Drp1, Opa1, Mfn1, Mfn2, and Mff) in chicken spleen lymphocytes.

The findings from the qRT PCR analysis method revealed the relative mRNA expression levels of energy metabolism-related genes, as depicted in Figure 6. In the NH_4_Cl group, a significant decrease in the relative mRNA expression of HK1, HK2, PFK, LDHA, LDHB, ACO2, and SDHB was observed when compared to the control group (*P* < 0.001). Conversely, in the Lut group, the relative expression of HK1 and HK2 mRNA exhibited an increase compared to the NH_4_Cl group, although the difference was not statistically significant (*P* > 0.05). The expression of LDHA mRNA exhibited a significant increase (*P* < 0.01), whereas the expression of LDHB, PFK, ACO2, and SDHB mRNA demonstrated a significant increase (*P* < 0.001). Lut effectively mitigated the disruption of energy metabolism induced by ammonia exposure in chicken lymphocytes.

### The Effect of Lut on the miR-155-5p/PI3K/AKT Signaling Pathway After Ammonia Exposure

**Figure 7:**
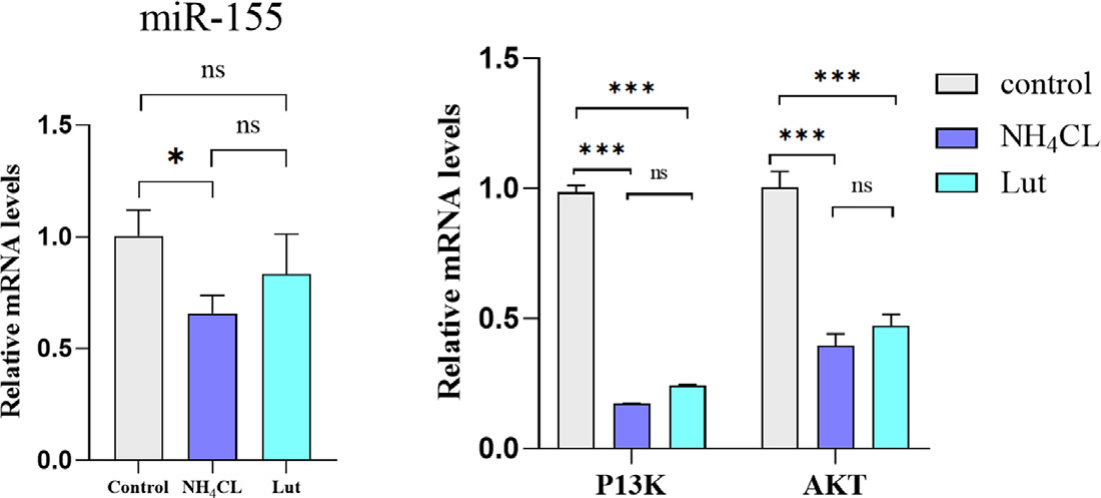
Results of qRT-PCR for miR-155-5p/PI3K/Akt pathway in chicken spleen lymphocytes.

To gain a deeper comprehension of the impact of Lut on the detrimental effects of ammonia exposure on chicken spleen lymphocytes, we employed qRT PCR to assess the mRNA expression of the miR-155-5p/PI3K/AKT signaling pathway, as depicted in Figure 7. In the NH_4_Cl group, a noteworthy decrease in the relative mRNA expression levels of PI3K and AKT (*P* < 0.001) was observed in comparison to the control group. Additionally, a significant reduction in the relative mRNA expression levels of miR-155-5p (*P* < 0.05) was also observed. In the Lut group, in comparison to the NH_4_Cl group, the relative expression levels of PI3K, AKT, and miR-155-5p mRNA exhibited a nonsignificant increase (*P* > 0.05), suggesting that ammonia stimulation activated the miR-155/PI3K/AKT pathway, leading to cellular damage. However, Lut demonstrated a mitigating effect on this damage by downregulating the expression of the miR-155-5p/PI3K/AKT signaling pathway.

## DISCUSSION

The detrimental impact of ammonia on poultry is prevalent, necessitating immediate attention to address the pressing issue of preventing and managing ammonia poisoning in contemporary intensive farming. Lut plays a crucial role in facilitating the release and enhancement of energy supply, thereby serving as a protective mechanism. The molecular mechanism underlying the protective effects of Lut on the energy metabolism pathway against damage caused by ammonia exposure was investigated through the assessment of various parameters including ATP content, ATPase activities, mitochondrial membrane potential, mitochondrial dynamics-related genes, energy metabolism, and the relative mRNA expression of the miR-155-5p/PI3K/AKT pathway in our experiment. Lut has the potential to mitigate the reduction in cell membrane potential induced by ammonia, as well as the impairment the content and activities of ATPase, dysregulation of mitochondrial dynamics gene expression, and alteration of metabolic pathway capacity. Furthermore, Lut can safeguard mitochondrial structure and alleviate structural harm, ultimately restoring energy metabolism equilibrium and exerting a protective influence against ammonia toxicity.

Mitochondrial respiration encompasses a sequential process occurring within the mitochondrial membrane, involving the establishment of a proton gradient, the transportation of electrons via the electron transport chain, and the phosphorylation of ADP to yield ATP (Carminita et al., 2021). The mitochondria play a crucial role in aerobic respiration, material metabolism, and energy conversion within cells (Greber and Ban, 2016). Disruptions in mitochondrial function can hinder ATP synthesis and decrease the absorption of mitochondrial metabolic substrates, thereby limiting the capacity of cells and organs to endure and recover from traumatic injury (Smolina et al., 2017). The process of ATP decomposition necessitates the participation of ATPase, a protein located on the mitochondrial membrane that is responsible for maintaining the electrochemical gradient of the inner mitochondrial membrane. This protein also plays a crucial role in maintaining ion homeostasis. Specifically, Na^+^/K^+^-ATPase functions as a sodium pump, responsible for regulating the concentrations of sodium (**Na^+^**) and potassium (**K^+^**), as well as controlling calcium (**Ca^2+^**) levels within the mitochondria (Shattock et al., 2015). Additionally, Ca^2+^-ATPase is responsible for transporting Ca^2+^ from the cytoplasm into the mitochondria, thereby ensuring the maintenance of Ca^2+^ homeostasis (Leijding et al., 2023). Mitochondrial damage has been demonstrated to have an impact on the synthesis of ATPase (O'Malley et al., 2020). This reduction in ATPase activity can consequently disrupt intracellular energy metabolism. Notably, investigations have revealed that infection with *Mycoplasma gallisepticum* induces a decline in ATPase activity, thereby leading to dysfunction in energy metabolism (Ishfaq et al., 2020). Furthermore, excessive levels of ammonia gas have been found to cause a decrease in ATPase activity in both the spleen of chickens (Li et al., 2021) and the heart of pigs (Cheng et al., 2022). The preservation of cellular homeostasis relies heavily on the integrity of mitochondrial quantity and structure. The mitochondrial membrane becomes permeable, leading to a decrease in mitochondrial transmembrane potential and subsequent mitochondrial dysfunction (Chen et al., 2020b). In the present study, the examination of lymphocytes exposed to ammonia revealed alterations in mitochondrial structure, rupture of mitochondrial cristae, and a reduction in mitochondrial quantity. Lut has been shown to enhance the levels and functions of ATPase, mitigate the deterioration of mitochondrial membrane potential, and safeguard against the structural impairment of mitochondria induced by ammonia-induced damage.

Mitochondria exhibit a high degree of dynamism as they undergo continuous fusion and division processes in order to adapt their shape in response to diverse stimuli and support energy metabolism. The proteins Mfn1, Mfn2, and Opa1 play a crucial role in preserving the morphology and structure of the cristae (Yapa et al., 2021). Opa1 plays a role in and sustains the process of mitochondrial fusion, thereby facilitating the occurrence of mitochondrial fusion and holding substantial importance in safeguarding the structure and functionality of mitochondria (Milanowski et al., 2022). Drpl and Mff are implicated in mitochondrial fission (Hu et al., 2021). Mitochondrial fusion and division are fundamental cellular processes that are indispensable for preserving the structure, abundance, and functionality of mitochondria (Meyer et al., 2017). The disruption of mitochondrial fusion and division occurs when the organism is stimulated by the external environment (Chan, 2020). This disruption can result in the breakage of mitochondrial cristae and the formation of mitochondrial vacuoles due to excessive division, while excessive fusion can lead to elongation of mitochondrial tubules (Mahecic et al., 2021). In the case of excessive ammonia exposure, mitochondrial dynamics imbalance and mitochondrial fission were observed in the chicken bursa of Fabricius. This was attributed to the upregulation of Drpl and Mff, as well as the downregulation of Opa1, Mfn1, and Mfn2 (Shah et al., 2020). According to Gao et al. (2022), exposure to PM_2.5_ resulted in an upregulation of Drp1 expression, a downregulation of Opa1 and Mfn1 expression, and the promotion of mitochondrial fission in the lung tissue of mice (Han et al., 2017). found that lead poisoning inhibited Mfn1, Mfn2, and Opa1 in chicken kidney, leading to an imbalance in mitochondrial dynamics and the occurrence of mitochondrial fission. Similarly, in human neuroblastoma cells (SH-SY5Y), Yang et al. (2020) observed that methamphetamine increased the level of Drp1, thereby inducing mitochondrial fission. In our experimental study, the findings from ultrastructural pathological observation revealed the presence of mitochondrial swelling and rupture of mitochondrial cristae. Given the strong association between mitochondrial structural damage and mitochondrial dynamic disorders, we proceeded to assess the expression levels of Drp1, Opa1, Mfn1, Mfn2, and Mff in lymphocytes. It was observed that Lut effectively suppressed the upregulation of Drp1 and Mff in lymphocytes following exposure to ammonia, while promoting an increase in the expression of Opa1, Mfn1, and Mfn2. This study examines the impact of ammonia exposure on the mitochondrial dynamics of chicken spleen lymphocytes, leading to mitochondrial fission. The administration of Lut demonstrates its ability to regulate the expression of genes involved in mitochondrial fusion and division, thereby promoting mitochondrial fusion and inhibiting mitochondrial fission.

Mitochondrial dysfunction disrupts cellular energy metabolism and hampers normal homeostasis (Wang et al., 2016). Furthermore, mitochondria play a crucial role in energy metabolism, as they undergo numerous redox reactions and serve as the primary site for material metabolism and energy generation. Mitochondria fulfill the function of serving as “energy providers” within cells (Heidari, 2019; Shi et al., 2023). The enzymes HK, PFK, PK, and LDH have the ability to catalyze intracellular glycolysis reactions and play a significant role in the regulation of glucose metabolism (Luo et al., 2022). The activity of HK has been found to be linked to hypoxia-induced autophagy in mouse cardiomyocytes (Li et al., 2022). SDH, an enzyme situated in the inner mitochondrial membrane, serves as a connection between oxidative phosphorylation (**OXPHOS**) and the electron transfer process (Moosavi et al., 2020). The presence of the noxious gas H_2_S has the potential to diminish the expression of AMPK, HK1, PFK, LDHA, and LDHB, thereby resulting in hepatic energy dysfunctions in chickens (Guo et al., 2019). Taurine possesses the ability to mitigate the decline in SDH activity, mitochondrial membrane potential, mitochondrial swelling, and ATP depletion induced by ammonia toxicity (Niknahad et al., 2017). This finding provides evidence that taurine can ameliorate mitochondrial dysfunction and energy metabolism disorders in the liver and brain of mice under the influence of ammonia toxicity. In the present study, it was observed that Lut supplementation resulted in an upregulation of energy metabolism-associated genes (HK1, HK2, LDHA, LDHB, PFK, PK, SDHB, and ACO2) in chicken spleen lymphocytes subjected to ammonia exposure. These findings suggest that Lut possesses the potential to mitigate energy metabolism disturbances induced by ammonia exposure.

Mitochondria play a crucial role in various physiological processes and serve as regulators of diverse signaling pathways. One such pathway is the PI3K-AKT pathway, which has been identified as a mitochondrial regulatory pathway (Sun et al., 2022). The activation of the PI3K/AKT signaling pathway in the cardiac tissue of pigs has been observed upon exposure to ammonia (Cheng et al., 2022). In the context of methamphetamine-induced cell apoptosis and autophagy, Lut has been found to exert inhibitory effects on the PI3K/AKT pathway, thereby attenuating these cellular processes (Tan et al., 2020). Furthermore, miR-155-5p has been identified as a pivotal miRNA involved in the regulation of the PI3K/AKT signaling pathway (Duan et al., 2018; Miao et al., 2022). In the present study, it was observed that Lut exhibited a tendency to enhance the expression of miR-155-5p/PI3K/AKT in lymphocytes following exposure to ammonia, although the observed effect did not reach statistical significance. Conversely, Lut demonstrated a notable capability to diminish the occurrence of autophagy and apoptosis in lymphocytes subsequent to ammonia exposure. These findings suggest that the miR-155-5p/PI3K/AKT signaling pathway may not be a pivotal mechanism underlying the protective effects of Lut.

## CONCLUSIONS AND FUTURE PERSPECTIVE

In our experimental study, it was observed that Lut effectively mitigates the morphological impairment of mitochondria caused by ammonia poisoning, as well as the reduction in mitochondrial membrane potential, ATP content, and ATPase activities following exposure of lymphocytes to ammonia. Furthermore, Lut demonstrates the ability to enhance mitochondrial fusion and suppress mitochondrial fission in spleen lymphocytes subsequent to ammonia exposure, thereby promoting equilibrium in energy metabolism and conferring protection against the toxic effects of ammonia.

Lut demonstrates the capacity to safeguard cell morphology and enhance cell energy supply in the presence of ammonia poisoning. As a feed additive, Lut exhibits considerable promise and holds substantial potential for implementation in animal husbandry. Subsequent investigations should aim to elucidate the underlying mechanism through which Lut counteracts ammonia poisoning, identify more efficacious targets, and establish a foundational framework for the subsequent development of Lut-based feed additives.
